# Mitochondrial DNA oxidation, methylation, and copy number alterations in major and bipolar depression

**DOI:** 10.3389/fpsyt.2023.1304660

**Published:** 2023-12-14

**Authors:** Deniz Ceylan, Bilge Karacicek, Kemal Ugur Tufekci, Izel Cemre Aksahin, Sevin Hun Senol, Sermin Genc

**Affiliations:** ^1^Affective Laboratory, Koç University Research Center for Translational Medicine, Istanbul, Türkiye; ^2^Department of Psychiatry, Koç University Hospital, Istanbul, Türkiye; ^3^Izmir Biomedicine and Genome Center, Genç Lab, Izmir, Türkiye; ^4^Brain and Neuroscience Research and Application Center, Izmir Demokrasi University, Izmir, Türkiye; ^5^Vocational School of Health Services, Izmir Democracy University, Izmir, Türkiye; ^6^Graduate School of Health Sciences, Koç University, Istanbul, Türkiye

**Keywords:** mtDNA, depression, methylation, epigenetics, oxidation, mitochondrial copy number

## Abstract

**Background:**

Mood disorders are common disabling psychiatric disorders caused by both genetic and environmental factors. Mitochondrial DNA (mtDNA) modifications and epigenetics are promising areas of research in depression since mitochondrial dysfunction has been associated with depression. In this study we aimed to investigate the mtDNA changes in depressive disorder (MDD) and bipolar disorder (BD).

**Methods:**

Displacement loop methylation (D-loop-met), relative mtDNA copy number (mtDNA-cn) and mtDNA oxidation (mtDNA-oxi) were investigated in DNA samples of individuals with MDD (*n* = 34), BD (*n* = 23), and healthy controls (HC; *n* = 40) using the Real-Time Polymerase Chain Reaction (RT-PCR). Blood samples were obtained from a subset of individuals with MDD (*n* = 15) during a depressive episode (baseline) and after remission (8th week).

**Results:**

The study groups exhibited significant differences in D-loop-met (*p* = 0.020), while relative mtDNA-cn and mtDNA-oxi showed comparable results. During the remission phase (8th week), there were lower levels of relative mtDNA-cn (*Z* = −2.783, *p* = 0.005) and D-loop-met (*Z* = −3.180, *p* = 0.001) compared to the acute MDD baseline, with no significant change in mtDNA-oxi levels (*Z* = −1.193, *p* = 0.233).

**Conclusion:**

Our findings indicate significantly increased D-loop methylation in MDD compared to BD and HCs, suggesting distinct mtDNA modifications in these conditions. Moreover, the observed alterations in relative mtDNA-cn and D-loop-met during remission suggest a potential role of mtDNA alterations in the pathophysiology of MDD. Future studies may provide valuable insights into the dynamics of mtDNA modifications in both disorders and their response to treatment.

## Introduction

Mood disorders, including major depressive disorder (MDD) and bipolar disorder (BD), are widespread and disabling psychiatric disorders (1). Patients with mood disorders manifest symptoms that arise from a complex interplay of genetic and environmental factors (2–4). Despite a plethora of findings implicating structural and functional alterations across various levels, ranging from microstructural and molecular pathways to neural networks, the understanding of the mechanisms underlying depression remains scarce (4). Recent evidence shows that mood disorders are linked to several mechanisms, including epigenetic regulation and oxidative stress, which can trigger diverse modifications in the genomic material, such as DNA methylation or oxidation (3, 5, 6).

Epigenetic regulation includes mechanisms that control gene expression without any change in DNA nucleotide sequence. A growing number of reports indicates that epigenetic mechanisms, such as DNA methylation, histone modifications, and non-coding RNAs may exert critical roles in the pathogenesis of mood disorders, as well as in the response to pharmacological interventions (3, 5, 7, 8). Among the epigenetic mechanisms, DNA methylation is the most extensively studied in mood disorders, which involves the addition of methyl groups to the DNA molecule. DNA methylation alterations have frequently been shown in patients with depression (9). In addition to methylation changes, DNA is also susceptible to oxidation by free radicals, leading to oxidation-induced DNA damage. Previous evidence supports that oxidation-induced DNA damage exists in the pathogenesis of depression (10–13). However, the findings are based solely on modifications of nuclear genetic material including nuclear DNA and RNAs.

Mitochondria are semi-autonomous organelles that contain their own, circular, maternally inherited, and double-stranded (i.e., heavy and light strands) mitochondrial DNA (mtDNA) and serve as a major energy supply of the human body. The mtDNA encodes 13 polypeptides belonging to electron transport chain complexes, 22 transfer RNAs, and 2 ribosomal RNAs, and contains a non-coding region, which includes a displacement loop (D-loop) (14, 15). Alterations in mtDNA may lead to changes in the expression of mitochondrial genes, affecting mitochondrial functioning and bioenergetic regulation of the body, resulting in mitochondrial dysfunction (16). Mitochondrial dysfunction has been identified as one of the key mechanisms underlying various aspects of depression, such as psychomotor symptoms and neurocognitive abnormalities, as well as early aging (17, 18). Previous research has reported abnormal mitochondrial morphology and altered levels of mitochondrial metabolites, genes, or proteins in both MDD and BD (19, 20) and suggests similar mitochondrial dysfunctions between these disorders (21–23). Despite mtDNA being more susceptible to genomic modifications, such as methylation and oxidation than nuclear DNA (24, 25), the recognition of mtDNA modifications, including mtDNA copy number (mtDNA-cn), oxidation (mtDNA-oxi), and methylation, has only recently been gaining attention in psychiatric research.

Recently, mtDNA methylation has emerged as a research topic, as methylation was previously thought to be absent in the mitochondrial genome. Although diverse types of epigenetic modifications have been shown in mtDNA, the major modifications include mtDNA methylation and hydroxymethylation (24). mtDNA methylation patterns show associations with several clinical factors including aging (26, 27), cardiovascular diseases (28), obesity (29), and neurodegenerative diseases (16), as well as environmental factors such as maternal smoking (30) and air pollution (31). The study of mitochondrial epigenetics in psychiatric disorders has recently gained attention. In the sole investigation of mtDNA methylation in individuals with major depressive disorder, results showed no difference in methylation levels in the mitochondrial D-loop region. However, there was a decrease in methylation in the PGC alpha region and an increase in mtDNA copy number (32).

mtDNA copy number is a measure of the number of mitochondrial genomes per cell, is considered a proxy measure for mitochondrial biogenesis, and has repeatedly been associated with mood disorders (33). Substantial data suggest alterations in mtDNA copy number in both MDD and BD (32, 34–39). On the other hand, a very limited number of studies focused on oxidation of mtDNA, although increased oxidation to nuclear DNA has repeatedly been shown in depression (11, 40, 41). Furthermore, mtDNA is three times more susceptible to the effects of free radicals than nuclear DNA, as it is located near by-products of oxidative phosphorylation and lacks protection from histones (42). Two clinical studies reported increased mtDNA damage in peripheral samples of patients with unipolar depression (13, 39), and a post-mortem study showed a significant decrease in brain slices of patients with BD (43).

mtDNA modifications might be a promising and novel area for depression research. In the current study, to further address the potential involvement of mtDNA changes (copy number, oxidation, and methylation) in depression pathogenesis, we quantified relative mtDNA-cn, mtDNA-oxi, and DNA methylation levels in the D-loop regulatory region (D-loop-met) in peripheral blood cells from individuals with MDD and BD and healthy individuals. In addition, we measured the mtDNA changes in a group of MDD, after resolution of depressive symptoms in the 8th week.

## Methods

This is a case control study conducted using blood samples provided by 23 acutely depressed patients with BD, 34 acutely depressed patients with MDD, and 40 healthy volunteers aged between 18 and 45 years. A subset of the MDD sample (*n* = 15) also provided blood samples in remission status at the 8th week after their inclusion in the study. All blood samples had been collected for a previous study of which findings were published (12). However, only 105 of 180 samples from the previous study were available for the current study, as the rest were consumed during the previous study. Additionally, we excluded 8 samples from participants with BD due to the small sample size of the group, which was insufficient for statistical analysis. Each participant gave informed consent for the samples to be archived and used in new studies. The current study was approved by the local ethics committee (2022.091.IRB2.016). All procedures were conducted in accordance with the provisions of the Declaration of Helsinki.

### Study subjects and data collection

Patients diagnosed with MDD or BD were recruited for this study from the Department of Psychiatry at Dokuz Eylul University in Izmir, Turkey. The study included a total of 57 patients, and alongside them, an HC group consisting of 40 healthy volunteers who responded to recruitment announcements was also recruited. The diagnosis of MDD or BD, as well as that of the HC group, was confirmed using the Structured Clinical Interview for DSM-IV-TR (SCID-I), administered by experienced clinicians. All participants underwent clinical rating scales, including the Hamilton Depression Rating Scale (44) and the Young Mania Rating Scale, administrated by experienced clinicians (45).

Participants with MDD or BD who scored below 18 on the 17-item Hamilton Depression Rating Scale or above 7 on the Young Mania Rating Scale were excluded from the study. Additionally, all participants completed the Self-report Perceived Stress Scale, Hypomania Checklist, and Healthy Lifestyle scales. Patients with any medical comorbidity or significant abnormalities in routine blood and urine tests, as well as those with any psychiatric comorbidity, were excluded from the study. For the healthy control group, exclusion criteria included the presence of any medical problems, known medical diagnoses, a history of psychiatric diagnoses, or a family history of depressive disorders, bipolar disorders, psychotic disorders, or neurodevelopmental disorders in first-degree family members. Pregnancy or breastfeeding and the use of any supplements, such as antioxidants, or any substance use disorders other than tobacco were exclusion criteria for all participants (12).

Since treatment response can usually be observed after 4–6 weeks, patients with MDD and BD were assessed in the 8th week to allow ample time for symptomatic remission (46). Remission was defined as not meeting the diagnostic criteria for depression in the SCID-I Interview during the 8th week and was justified by a total HDRS score of <7.

### Materials and methods

A total of 10 ml of venous blood was collected in EDTA-coated tubes from each patient or healthy individual after an overnight fast (10 h) between 8:00 and 10:00 a.m. Whole blood samples were then stored at −80°C. DNA from each sample was extracted from whole blood samples using DNeasy Blood and Tissue kits (Qiagen; Hilden, Germany), following the manufacturer's instructions. The amount and purity of DNA samples were measured using a Nanodrop 2000 spectrophotometer (Thermo Scientific). The DNA samples were stored at −20°C until they were used for PCR analysis, which included copy number, oxidation, and D-loop methylation analysis. All selected mtDNA primers in the PCR analysis are given in the Supplementary Table.

#### Assessment of D-loop-met

Bisulfite conversion and methylation-specific PCR processes were performed as previously described (32). This technique is based on the metDNA/unmetDNA ratio measurements. For mtDNA linearization, genomic DNA was treated with BamHI before bisulfite conversion (47). Bisulfite conversion of purified DNA was performed using the EpiJET Bisulfite conversion kit (Thermo Scientific, Korea) according to the manufacturer's protocol. In the bisulfite reaction, all unmethylated cytosines are converted to uracils, while methylated cytosines remain unchanged. MSP was performed using two specific primer pairs, M primers for methylated DNA (metDNA) and U primers for unmethylated DNA (unmetDNA) in the mitochondrial D-loop region (29). 10 ng of bisulfite-converted DNA with IQ SYBR Green Supermix (Bio-Rad Laboratories) was used. DNA methylation status was evaluated according to the following formulas: ΔCt = CtmetDNA – CtunmetDNA and metDNA/unmetDNA ratio 2^−ΔCt^.

#### Assessment of relative mtDNA-cn

The mtDNA-cn was determined by the ratio of mtDNA to the single-copy nuclear DNA, as described previously (32, 48). The quantities of mtDNA and nuclear DNA were represented by the threshold cycle (Ct) of three mitochondrial genes, Cytochrome b (*CYTB*), NADH dehydrogenase 1 (*ND1*), NADH dehydrogenase 4 (*ND4*), and a single-copy nuclear gene, pyruvate kinase (*PK*), respectively, using quantitative polymerase chain reaction (qPCR) analyses. The mixtures of qPCR for both CYTB and PK were identically composed of 20 ng of genomic DNA and the IQ SYBR Green Supermix in 10 μL reaction samples, except for each primer set. Each sample was measured in triplicate (all standard deviations [SD] of the duplicate threshold cycle were < 0.7). The mtDNA-cn was calculated using the following formula: ΔCt = Ct mitochondrial gene – Ct PK, and consequently 2^−Δ*Ct*^ calculation. Consequently, ΔΔCt = ΔCt sampe-ΔCt average of controls, and finally 2^−ΔΔ*Ct*^ calculation was used to obtain relative mtDNA-cn. A composite relative mtDNA-cn was calculated using arithmetic means of CYTB, ND1 and ND4 copy numbers, as previously done (49).

#### Assessment of mtDNA-oxi

mtDNA oxidation was determined using a qPCR technique as previously described (13). This technique is based on the principle that DNA lesions on a template strand can block DNA polymerase, thereby causing reduced amplification of the target sequence. Because only undamaged DNA templates are amplified, the longer the amplicon, the greater the chance of lesion. All reactions are measured in duplicate. The results are presented as the number of lesions per 10,000 base pairs. The number of lesions per fragment was calculated using the formula number of lesions = –ln 2^(Δ*Ctshort*−Δ*Ctlong*)^. Ct short is the difference between the mean threshold cycle of the sample and the reference obtained using primers for 100-bp amplicons, whereas Ct long is the mean threshold cycle of the samples vs. 1,000-bp is the difference between the reference obtained using primers for amplicons. The mean CT of healthy controls was used as reference. A composite mtDNA-oxi was calculated using arithmetic means of ND1 and NADH dehydrogenase 5 (*ND5*) genes.

### Statistical analysis

Statistical analyses were performed using the IBM SPSS Statistics 28.0 (Chicago IL, USA) software. The GraphPad was used to create graphs. The study groups (i.e., MDD, BD, and HC) were compared for sociodemographic data, clinical measures, and results of the PCR analysis. Categorical data such as sex, diagnosis, and smoking status were presented as percentages, and compared among groups using the Chi-square test. Continuous data were presented as mean and standard deviations or medians, and minimum-maximum values regarding its Gaussian distribution. The Gaussian distribution of continuous data was checked using quantile-quantile plots, distribution of data in histograms, and verified with the Shapiro-Wilk test.

Univariate Analysis of Covariance or Quade Non-parametric Analysis of Covariance models were used due to non-Gaussian distribution of data. ANCOVA models included age, sex, body mass index, and smoking status as covariates. For the follow-up (remission state) measurements, the Wilcoxon Test was used. Spearman correlation tests were used to determine correlations among demographic and clinical variables and PCR results. Linear regression models were applied to identify the associations between main findings and certain variables: sociodemographic variables (i.e., age, sex, body mass index, and smoking status), clinical variables [i.e., type of depression (MDD or BD), duration of current episode], scale scores (i.e., Hamilton Depression, Young Mania, Perceived Stress, and Healthy Lifestyle Scales), medications (i.e., antidepressants, mood stabilizers, and antipsychotics). A significance level of 0.05 was employed, and to account for multiple comparisons, a Benjamini-Hochberg (BH) correction with a false discovery rate (FDR) of 0.1 was applied (50).

## Results

Demographic and clinical characteristics of MDD, BD, and HC are summarized in Table 1. Mean age of the BD group was significantly higher than that of the HC group (MDD vs. BD, *p* = 1; MDD vs. HC, *p* = 0.078; BD vs. HC, *p* = 0.036). The study groups did not differ in terms of the other sociodemographic variables (Table 1). In terms of clinical measures, the groups showed significant differences in mean scores of the Hamilton Depression, Young Mania, Hypomania Checklist, Healthy Lifestyle, and Perceived Stress scales (Table 1). Only 2 individuals in the BD group had BD Type II, while others had BD Type I disorder.

**Table 1:** Sociodemographic and clinical characteristics of the study groups.

	**MDD (*n* = 34)**	**BD (*n* = 23)**	**HC (*n* = 40)**	**Statistics**
Age	32.91 ± 8.73	34.09 ± 7.12	28.60 ± 8.23	***F*** **=** **4.18**, ***df*** **=** **2**, ***p*** **=** 0.018^*^
Sex (female %)	77.4%	68.2%	64.7%	*X*2 = 1.30, *df* = 2, *p* = 0.521^#^
Partnership status (couple %)	50%	36.4%	32.4%	*X*2 = 2.37, *df* = 2, *p* = 0.306^#^
Working status (employed %)	50%	72.7%	44.1%	*X*2 = 4.64, *df* = 2, *p* = 0.098^#^
Body mass index (kg/m^2^)	25.05 ± 4.82	26.07 ± 4.90	23.49 ± 4.04	*F* = 2.56, *df* = 2, *p* = 0.083^*^
Exercise (yes %)	20.6%	33.34%	47.1%	*X*2 = 5.33, *df* = 2, *p* = 0.070^#^
Smoking (non-smoker %)	38.2%	59.1%	66.7%	*X*2 = 5.96, *df* = 2, *p* = 0.051^#^
Alcohol (no alcohol %)	58.8%	54.5%	44.1%	*X*2 = 3.53, *df* = 2, *p* = 0.474^#^
Hamilton depression rating scale	21.15 ± 4.93	21.27 ± 11.08	0.56 ± 1.31	***F*** **=** **177.45**, ***df*** **=** **2**, ***p*** **<** 0.001^*^
Young Mania rating scale	1.50 ± 1.52	2.00 ± 1.98	0.22 ± 0.71	***F*** **=** **46.49**, ***df*** **=** **2**, ***p*** **=** 0.02^*^
Perceived stress scale	32.27 ± 6.90	31.44 ± 8.59	21.74 ± 7.43	***F*** **=** **19.51**, ***df*** **=** **2**, ***p*** **<** 0.001^*^
Hypomania checklist	14.11 ± 7.25	22.53 ± 7.38	16.84 ± 6.01	***F*** **=** **8.92**, ***d*** **=** **2**, ***p*** **<** 0.001^*^
Healthy lifestyle scale	96.85 ± 22.55	102.67 ± 27.86	134.83 ± 20.90	***F*** **=** **24.28**, ***df*** **=** **2**, ***p*** **<** 0.001^*^

Continuous variables were presented as mean ± sd; categorical variables were presented as percentages.

* ANOVA.

# Chi-square. Bold features mean statistically significant results.

In the *post-hoc* analysis, both the MDD and BD groups had higher scores in the Hamilton Depression (*p* < 0.001; *p* < 0.001), Young Mania (*p* = 0.018; *p* = 0.002), Healthy Lifestyle (*p* < 0.001; *p* < 0.001), and Perceived Stress (*p* < 0.001; *p* < 0.001) scales in comparison to the HC group. Concerning the Hypomania Checklist, the BD group exhibited significantly higher scores compared to both the MDD and HC groups (*p* < 0.001; *p* = 0.014), whereas the MDD and HC groups presented comparable scores (*p* = 0.368).

Thirty-two percentage of the individuals with MDD (*n* = 11) and 17% of the individuals with BD (*n* = 4) were drug-free, while others were receiving at least one medication. Forty-four percentage (*n* = 15) of the individuals with MDD were receiving one, 24% (*n* = 8), two antidepressant treatments. Seventeen percentage of individuals with BD (*n* = 4) were receiving one, 48% (*n* = 11), two, and 17% (*n* = 4), three or more medications. Only 9% (*n* = 2) of individuals with BD had BD type II, and others had BD type I. mtDNA changes of 17 individuals with MDD (50%) and 8 individuals (35%) with BD were re-evaluated in remission at the 8th week.

### mtDNA modifications among the study groups

#### Relative mtDNA-cn

**Figure 1:**
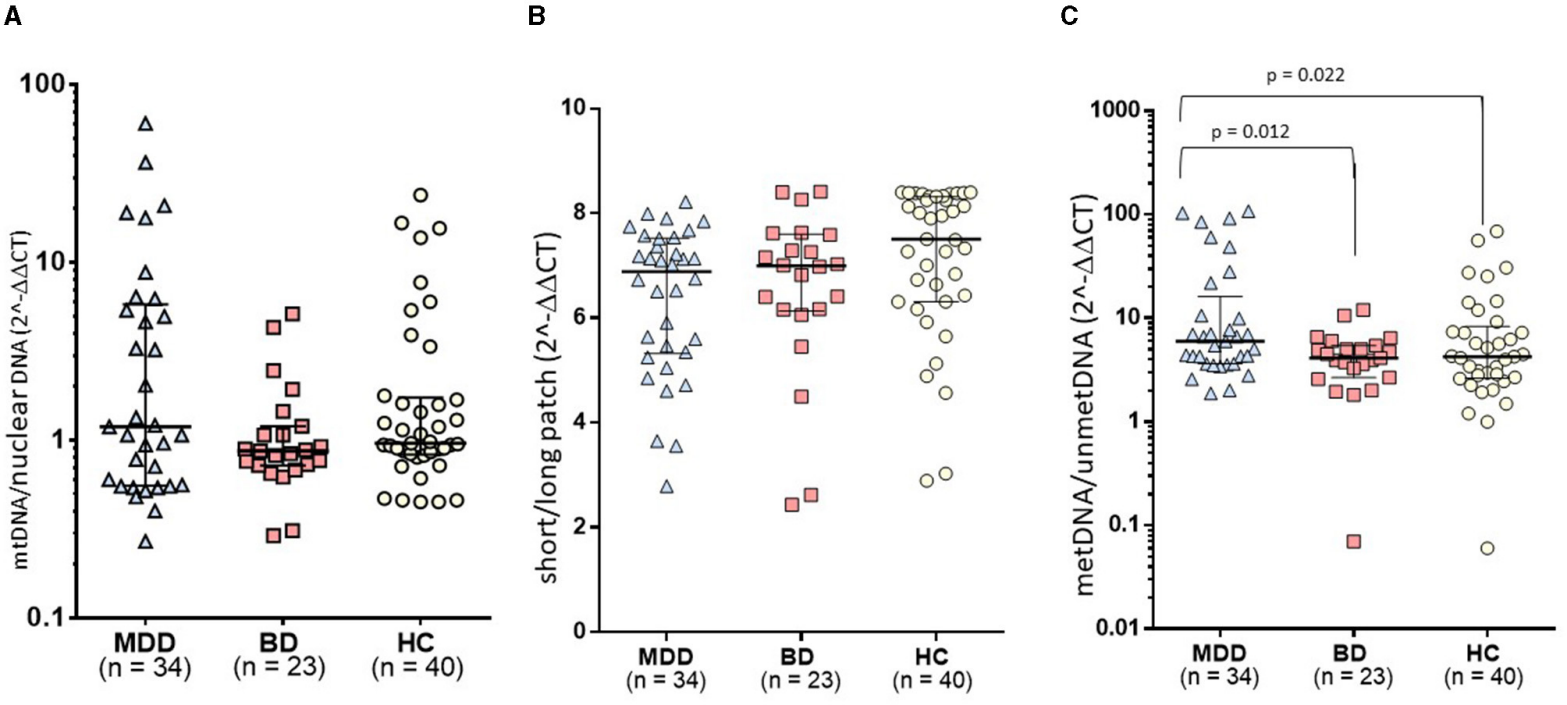
Comparison of mtDNA variations among study groups: **(A)** The relative mtDNA-cn composite, calculated using the average of copy numbers of 3 mitochondrial genes (i.e., CYTB, ND1, and ND4), was comparable among the groups. **(B)** The mtDNA-oxi composite value, as measured using average of oxidation levels of ND1 and ND5 genes, was comparable among groups. **(C)** The study groups exhibited a significant difference in the D-loop methylation levels (*F* = 4.068, *df* = 2, *p* = 0.020). In *post-hoc* comparisons, the MDD group presented significantly higher levels of mtDNA D-loop methylation compared to both HC (*p* = 0.022) and BD groups (*p* = 0.012), while the BD group was comparable with the HC group.

**Figure 2:**
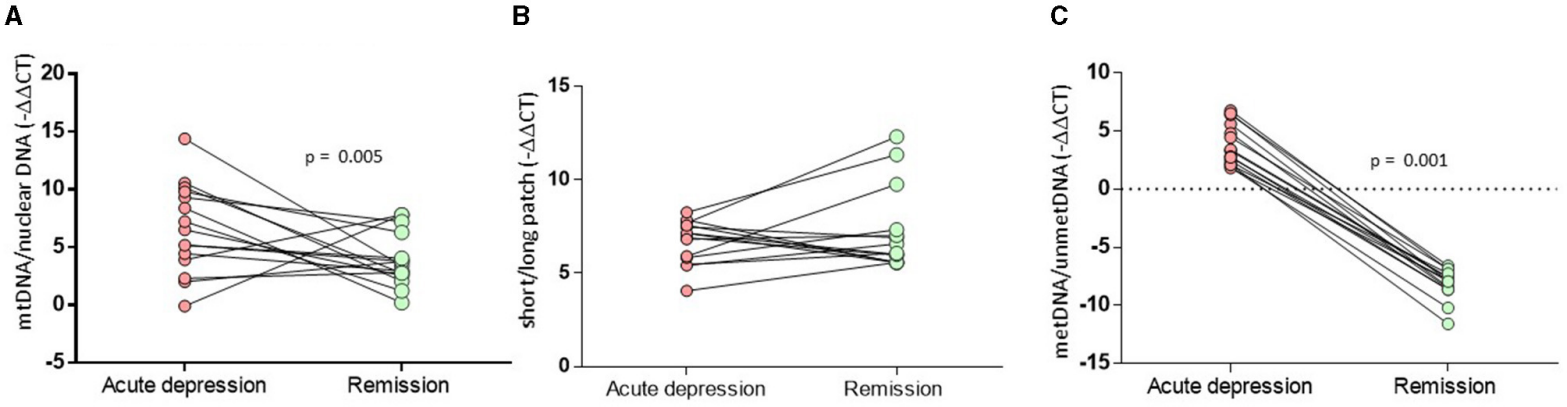
Comparison of MtDNA alterations in MDD patients between the acute and remitted phases. **(A)** The composite relative mtDNA-cn displayed a significant decrease in the remission compared to acute MDD (*Z* = −2.78, *p* = 0.005). **(B)** The composite mtDNA-oxi did not display a significant change in remission compared to acute MDD (*Z* = −1.193, *p* = 0.233). **(C)** The D-loop-met levels displyaled significantly decreased methylation levels in remission compared to those on acute depression (*Z* = −3.180, *p* = 0.001).

A relative mtDNA-cn composite, calculated using the average of copy numbers of 3 mitochondrial genes (i.e., CYTB, ND1, and ND4), was comparable among the groups, as illustrated in Figure 1 (*F* = 0.18, *df* = 2, *p* = 0.181). The copy numbers for each gene are moderately correlated (ND1-ND4: *r* = 0.536, *p* < 0.001; ND1-CYTB: *r* = 0.540; *p* < 0.001; ND4-CYTB *r* = 0.687; *p* < 0.001). ND4 and CYTB copy numbers are strongly correlated with the relative mtDNA-cn composite (*r* = 0.901, *p* < 0.001; *r* = 0.805, *p* < 0.001), while ND1 is moderately correlated (*r* = 0.668; *p* < 0.001). The copy numbers, as determined by CYTB, ND1, or ND4 genes separately, did not show any significant differences among the study groups, as illustrated in Table 2. On the other hand, during the follow-up period, both the composite relative mtDNA-cn displayed a significant decrease in the remission compared to acute MDD (*Z* = −2.78, *p* = 0.005), as illustrated in Figure 2. The remitted participants with MDD (*n* = 15) had lower relative mtDNA-cn than HCs [MDD: 0.68 (0.39–1.74); HC: 1.03 (0.93–1.13); *F* = 6.102, *p* = 0.017].

**Table 2:** The expression levels of mitochondrial genes and composite scores for copy number and oxidation.

	**MDD (*n* = 34)**	**BD (*n* = 23)**	**HC (*n* = 40)**	Statistics^*^
mtDNA-cn (composite)	322.93 (74.08–10,091.89)	235.43 (79.95–110.34)	268.62 (122.03–3,610.04)	*F* = 1.53, *df* = 2, *p* = 0.220
Relative mtDNAcn (composite)	1.20 (0.27–60.48)	0.87 (0.29–5.12)	0.96 (0.45–23.94)	*F* = 0.18, *df* = 2, *p* = 0.181
Relative CYTB-cn	0.94 (0.07–24.43)	0.74 (0.28–7.87)	0.74 (0.36–10.05)	*F* = 0.74, *df* = 2, *p* = 0.480
Relative ND1-cn	1.07 (0.34–3.41)	1.07 (0.34–2.41)	1.13 (0.53–3.73)	*F* = 0.27, *df* = 2, *p* = 0.761
Relative ND4-cn	0.87 (0.22–154.46)	0.77 (0.25–8.03)	0.91 (0.03–70.60)	*F* = 2.56, *df* = 2, *p* = 0.083
**mDNA-oxi**				
**ND1**	7.06 (0–7.93)	7.12 (0–8.21)	7.64 (0–8.23)	*F* = 3.12, *df* = 2, *p* = 0.049
ND5	6.90 (2.80–8.60)	6.5 (2.80–8.60)	7.88 (2.10–8.60)	*F* = 2.41, *df* = 2, *p* = 0.096

The gene expressions were presented as median (minimum-maximum) values. Composite scores are calculated as arithmetic means. All comparisons were adjusted for age, sex, body mass index and smoking status.

* Quade Non-parametric ANCOVA. Bold features mean statistically significant results.

#### mtDNA oxi

The mtDNA-oxi composite value, as measured using average of oxidation levels of ND1 and ND5 genes, show a significant difference among the study groups (*F* = 3.230, *df* = 2, *p* = 0.044), however, the significance disappeared after a Benjamini Hochberg correction. The mtDNA-oxi levels for ND1 and ND5 genes are moderately correlated (ND1-ND5: *r* = 0.690, *p* < 0.001), and both genes are strongly correlated with the mtDNA-oxi value (*r* = 0.840, *p* < 0.001; *r* = 0954, *p* < 0.001). The levels of oxidation, as determined by the ND1 gene but not the ND5 gene, showed a significant difference between the groups, as shown in Table 2 (respectively, *F* = 4.621, *df* = 2, *p* = 0.012; *F* = 2.263, *df* = 2, *p* = 0.110). The oxidation level of ND1 gene is lower in the MDD group but not in the BD group, in comparison to the HC group (MDD vs. HC: *p* = 0.003; MDD vs. BD: ns; BD vs. HC: ns). Additionally, during the follow-up period, the composite mtDNA-oxi did not display a significant change in remission compared to acute MDD (*Z* = −1.193, *p* = 0.233), as illustrated in Figure 2.

#### D-loop-met

The study groups exhibited a significant difference in the D-loop-met levels (*F* = 4.068, *df* = 2, *p* = 0.020), as illustrated in Figure 1. In *post-hoc* comparisons, the MDD group presented significantly higher levels of mtDNA D-loop methylation compared to both HC (*p* = 0.022) and BD groups (*p* = 0.012), while the BD group was comparable with the HC group (*p* = 0.601). After excluding two samples that appeared to be outliers in Figure 1C, the difference in D-loop-met levels remained statistically significant (*F* = 3.277, *df* = 2, *p* = 0.042). The MDD group exhibited significantly higher levels of mtDNA D-loop-met in comparison to both the HC group (*p* = 0.044) and the BD group (*p* = 0.023), while the BD group showed similar levels to the HC group (*p* = 0.597). Regarding the D-loop-met, the Wilcoxon signed-rank tests indicated significantly decreased methylation levels in remission compared to those on acute depression (*Z* = −3.180, *p* = 0.001), as illustrated in Figure 2.

#### mtDNA modifications and clinical variables

In the whole sample, there was a moderate correlation between relative mtDNA-cn and D-loop-met (*r* = 0.423, *p* < 0.001), which remains significant despite BH correction. There were no significant correlations between mtDNA modifications and clinical measures in the BD and HC groups. However, in the MDD group, relative mtDNA-cn showed a significant negative correlation with the total score of the Perceived Stress Scale (*r* = −0.638, *p* < 0.001). Although there were positive correlations between the Healthy Lifestyle Scale scores and relative mtDNA-cn or mtDNA-oxi, these correlations were not significant after BH correction (Table 3).

**Table 3:** Correlations between mtDNA modifications and clinical variables.

		**Total population (*****n*** = **97)**			**MDD (*****n*** = **34)**			**BD (*****n*** = **23)**			**HC (*****n*** = **40)**		
		**mtDNA-cn**	**mtDNA-oxi**	**D-loop-met**	**mtDNA-cn**	**mtDNA-oxi**	**D-loop-met**	**mtDNA-cn**	**mtDNA-oxi**	**D-loop-met**	**mtDNA-cn**	**mtDNA-oxi**	**D-loop-met**
mtDNA-oxi	*r*	**0.227**	-	-	**0.618** ^**^	**-**	**-**	0.208	**-**	**-**	0.217	**-**	**-**
	*p*	**0.027**	-	-	**< 0.001**	**-**	**-**	0.352	**-**	**-**	0.332	**-**	**-**
D-loop-met	*r*	**0.423** ^**^	0.115	-	**0.462**	**0.381**	-	0.159	0.071	-	0.232	−0.099	-
	*p*	**< 0.001**	0.278	-	**0.007**	**0.029**	-	0.470	0.755	-	0.286	0.660	-
Age	*r*	−0.050	−0.086	−0.043	0.010	−0.049	0.036	0.164	0.049	−0.157	0.042	0.124	−0.189
	*p*	0.626	0.407	0.686	0.953	0.784	0.844	0.455	0.829	0.473	0.849	0.584	0.389
Body mass index	*r*	0.071	0.042	0.091	0.266	**0.345**	0.031	−0.064	−0.127	0.294	−0.192	−0.159	0.089
	*p*	0.490	0.683	0.385	0.128	**0.046**	0.864	0.770	0.573	0.173	0.381	0.481	0.686
Young Mania rating scale	*r*	0.162	0.082	0.128	**0.378**	0.236	0.321	−0.223	0.021	−0.256	−0.186	0.092	−0.206
	*p*	0.131	0.451	0.236	**0.027**	0.178	0.068	0.318	0.927	0.250	0.407	0.690	0.359
Hamilton depression rating scale	*r*	−0.024	−0.114	0.027	−0.008	0.053	−0.102	0.018	−0.140	−0.156	0.190	0.092	−0.242
	*p*	0.827	0.296	0.807	0.962	0.768	0.571	0.936	0.544	0.487	0.398	0.691	0.278
Hypomania checklist	*R*	−0.005	0.169	−0.024	0.052	0.215	0.123	−0.028	**0.515**	0.144	−0.074	0.204	0.044
	*p*	0.963	0.138	0.835	0.798	0.282	0.542	0.908	**0.024**	0.555	0.763	0.403	0.858
Perceived stress scale	*r*	−0.174	−0.140	0.007	–0.638^**^	–0.559^**^	−0.388	0.007	−0.066	−0.208	−0.064	0.062	−0.227
	*p*	0.135	0.232	0.949	**< 0.001**	**0.003**	0.050	0.979	0.796	0.406	0.800	0.806	0.364
Healthy lifestyle scale	*r*	0.153	0.138	−0.044	**0.402**	**0.468**	**0.406**	−0.180	−0.121	−0.118	−0.029	0.007	−0.443
	*p*	0.201	0.252	0.714	**0.042**	**0.016**	**0.039**	0.522	0.668	0.675	0.919	0.980	0.098

** Significant despite BH correction. Bold features mean statistically significant results.

In whole patients, a significant linear regression (*F* = 2.869, *p* = 0.025), including age, sex, body mass index and medication (medicated and unmedicated) along with depression type (MDD and BD), revealed that D-loop-met levels are associated with body mass index (*B* = 2.125, *t* = 2.387, *p* = 0.021, CI: 0.332–3.919), being medicated (*B* = −16.703, *t* = −2.037, *p* = 0.048, CI: −33.22 to −0.185), depression type (*B* = −14.94, *t* = −2.11, *p* = 0.040, CI: −29,204 to −0.676).

## Discussion

The study of mitochondrial modifications and mitoepigenetic pathways related to mood disorders is gaining increasing popularity. A new understanding of the mechanisms driving mitochondrial dysfunction in mood disorders including MDD and BD could be gained from mitoepigenetic research given the growing data showing mitochondrial dysfunction in these disorders. In this 8-week follow-up study including acutely depressive individuals with MDD and BD, our objective was to compare markers of mtDNA modifications among individuals with MDD, BD, and HCs. We also sought to investigate how these mtDNA modifications change during remission in MDD. The primary measures for mtDNA modifications were D-loop-met, relative mtDNA-cn, and mtDNA-oxi. Our findings revealed that the MDD group exhibited higher levels of D-loop-methylation compared to both the BD and HC groups. However, we did not observe significant changes in levels of relative mtDNA-cn and mtDNA-oxi among the study groups; rather, we noticed trends of change in these parameters. During the follow-up period, individuals with MDD showed significant decreases in levels of D-loop-methylation and mtDNA copy number after remission of acute depression.

Unlike nuclear DNA, which exists in only two copies per cell, mtDNA is present in multiple copies per cell, depending on the specific cell type (51). Cells with higher energy demands, such as the brain, heart, and skeletal muscle, tend to have higher mtDNA copies (52). MtDNA-cn, which represents the ratio of mitochondrial to nuclear DNA, is considered a surrogate measure of mitochondrial biogenesis and a crucial marker of mitochondrial health (33). In our study, we employed a PCR-based method to determine relative mtDNA-cn. We conducted RT-PCR to amplify the nuclear PK gene and three mitochondrial genes (i.e., ND1, ND4, and CYTB). Subsequently, the mtDNA copy numbers were calculated and are presented as the ratio of mtDNA to nuclear DNA (as shown in Table 2), in line with several studies reporting mtDNA copy numbers in terms of fold changes (53, 54) We also determined the relative mtDNA-cn, as depicted in figures and Table 2, employing the ΔΔCt formula. This method involves subtracting the average Ct values of control samples from the Ct values of each test sample, in accordance with previous literature that has employed the relative Ct method (55, 56).

Several studies have reported a significant increase in relative mtDNA-cn in individuals experiencing acute depressive episode (32, 35, 36, 38, 57, 58) and significant decreases in acute (53, 55, 59) or clinically stable individuals with BD (39, 60, 61) compared to healthy individuals. Furthermore, a previous study comparing MDD and BD reported a significant increase in relative mtDNA-cn in MDD and a significant decrease in BD type I (38). Additionally, studies are reporting decreased relative mtDNA-cn in BD type I and increased relative mtDNA-cn in BD type II (61). In our study, we focused on acutely depressed individuals with either MDD or BD. The BD group consisted of 21 BD type I patients and 2 BD type II patients. Drawing from the literature mentioned above, we formulated the hypothesis of decreased relative mtDNA-cn in BD and increased relative mtDNA-cn in MDD. Upon observation, we noted a rising trend in relative mtDNA-cn in MDD and a non-significant tendency toward decreased levels in BD. Considering the general trend observed in the studies mentioned earlier, our non-significant results may be attributable to a Type II statistical error. However, drawing firm conclusions is challenging, primarily due to the high variability in the data.

Even though our findings did not reveal significant differences in acutely depressed patients with BD or MDD in comparison to healthy individuals, there was a significant decrease in relative mtDNA-cn of individuals with MDD after the resolution of depressive symptoms in the 8th week (Figure 2). To the best of our knowledge, this is the first study indicating a decrease in mtDNA in response to remission status. In a longitudinal study conducted by Fernström et al. (62), it was found that relative mtDNA-cn did not differ during the 8-week follow-up of individuals with MDD (62). However, it is important to note that in this study, only 11 of the participants were in remission in the 8th week, while 22 participants were still unremitted. This indicates that the follow-up findings do not fully reflect the remission status of all the participants at that specific point in time. In contrast, our study involved blood sampling only from individuals with MDD who achieved remission (*n* = 15) in the 8th week, providing a focused perspective on the remitted group. Our finding may suggest that the tendency for an increase in copy number during the acute phase might be a reversible acute reaction. In the additional analysis, we observed that relative mtDNA-cn levels of remitted individuals with MDD are lower than those of the HC group. This finding is consistent with a previous study by Chang et al. (39), which showed significantly decreased levels of relative mtDNA-cn in patients with MDD who had stable treatments for at least 2 months and achieved remission, as indicated by low scores on the clinical global impressions scale. This suggests that individuals with MDD who are in remission may tend to have lower levels of relative mtDNA-cn. During acute depression, there might be an increase in mtDNA-in as a response to enhance mitochondrial functioning.

To our best knowledge, this is the first study to compare mtDNA oxidation levels in peripheral samples of patients with MDD and BD. Using PCR technique, we compared the amplification of short and long patches of the ND1 and ND5 genes, based on the principle that DNA lesions on a template strand can obstruct DNA polymerase (13). Although our findings presented a significant difference in mtDNA-oxi levels among study groups, this significance did not remain after adjusting for possible multiple comparison errors (FDR = 0.1). On the other hand, oxidation levels that were measured by ND1 gene revealed a significantly reduced oxidation in MDD compared to HC, even after adjustments for age, sex, body mass index, smoking status, and multiple comparison errors. Despite non-significant results, ND5 gene oxidation levels presented a similar trend to ND1. The significance on the edge might be caused by a type II error. Our findings may suggest a trend of decrease in mtDNA-oxi in MDD and BD, however further studies with larger sample sizes are required. On the other hand, no changes were observed between the acute depression and remission phases regarding mtDNA-oxi levels. Previous studies have reported increased mtDNA damage in peripheral blood mononuclear cells of MDD patients (13, 39), a post-mortem study revealed a significant decrease in brain slices from BD patients (43). Further data using more sophisticated techniques is necessary to elucidate the dynamics of mtDNA-oxi in both disorders.

The mitochondrial D-loop is a non-coding region of mtDNA that contains regulatory elements controlling the replication and transcription processes of mtDNA (63). Changes or mutations in the D-loop region can impact mitochondrial function and have been implicated in various diseases, including neurodegenerative disorders, mitochondrial disorders, and mood disorders. According to previous data, decreased methylation in the D-loop region of mtDNA is associated with ALS, Alzheimer's Disease, and Parkinson's Disease (16, 64–66). The only study that focused on D-loop-met in individuals with MDD found comparable D-loop-met levels between MDD and healthy individuals. In contrast, our findings revealed significantly higher mitochondrial D-loop-met in MDD than BD and HC, indicating a specific hypermethylation of D-loop region in MDD. Additionally, dramatic decrease in D-loop-met was observed by remission of a major depressive episode (Figure 2), suggesting a reversible increase in acute MDD. As far as we know, there is no previous data showing dynamics of D-loop-met in response to acute depressive episode or remission. Our finding indicates that patients with BD have relatively lower D-loop-met compared to patients with MDD. This may suggest the presence of a distinct mitochondrial background in MDD and BD, highlighting potential differences in mitochondrial functioning between MDD and bipolar depression.

Combining our findings, our data reveal a non-significant trend of increased mtDNA-cn and a significant increase in D-loop-met. These observations contrast with a previous study that reported an increase in mtDNA-cn but no change in D-loop-met levels in MDD, even though the same laboratory techniques were employed (32). The disparity in D-loop-met levels may be attributed to differences in the populations under investigation. In the study conducted by Chung et al. (32), the population was drawn from patients who regularly visited an outpatient psychiatric clinic, potentially including individuals in remission or with mild depression. In contrast, our study focused on acutely depressive patients with moderate or severe depression, as assessed by the Hamilton Depression Scale, which could have contributed to the observed decrease in D-loop-met levels. Our finding of a substantial decrease in D-loop-met levels during remission supports this hypothesis. Moreover, it's worth noting that our study included participants aged between 18 and 45, whereas the average age of the population in the previous study was around 47. This age difference could be a contributing factor to the variations observed in D-loop-methylation (D-loop-met) levels. Additionally, we deliberately excluded individuals in the perimenopausal period from our sample, while the mentioned study included them. The perimenopausal period is known to be associated with epigenetic aging (67). This difference in the composition of the study groups may have resulted in contradicting results between two studies. On the other hand, the disparity in mtDNA-cn results between two studies could be attributed to our relatively small sample size, as discussed above, as well as the differences in populations.

Additionally, both relative mtDNA-cn and D-loop-met tend to decrease by remission of MDD. These trends may indicate significant differences in relative mtDNA-cn changes between MDD and BD that could potentially be used as markers to distinguish the two disorders, especially during acute depression. Distinguishing MDD from BD is critical for early diagnosis and appropriate treatment because many individuals with BD are initially misdiagnosed with MDD, resulting in a delay of ~6 years for incorrect diagnosis and treatment (68). Further longitudinal studies following acute and remission phases would help understand dynamics of mtDNA variation in both MDD and BD.

In addition, our findings revealed a moderate correlation between D-loop-met and relative mtDNA-cn, which is supported by previous studies showing correlations between mtDNA D-loop-met and relative mtDNA-cn in human and mouse cell cultures (69, 70), blood samples (29, 31), and human placenta (71). Correspondingly, the dramatical decrease in D-loop-met was accompanied by decreased levels of relative mtDNA-cn by remission of a major depressive episode (Figure 2). Our data presented no significant correlations between mtDNA modifications and clinical measures in the BD and HC groups. On the other hand, both relative mtDNA-cn and D-loop-met levels showed moderate negative correlations with perceived stress. On the other hand, a significant linear regression analysis revealed that D-loop-met levels are influenced by body mass index, being medicated (negatively), and type of depression.

The study's strengths included strict inclusion and exclusion criteria for participants and rigorous statistical corrections for various confounders and multiple comparisons. The follow-up design of the study was also a significant strength as it allowed for controlling the effects of depressive symptoms and remission by considering individual lifestyle factors as confounding variables. Consequently, the analysis revealed significant decreases in both relative mtDNA-cn and D-loop-met in MDD patients during remission in the 8th week. These observed alterations in relative mtDNA-cn and D-loop-met during remission suggest a potential role of mtDNA in the pathophysiology of MDD. As a methodological strength, we enhanced our approach by calculating a composite copy number through the arithmetic mean of three genes (i.e., ND1, ND4, and CYTB). This approach was based on the literature, where most studies utilized the ND1 gene as the sole mitochondrial gene (37, 39, 54, 62, 72), while other studies employed CYTB, ND4, or ND5 genes (61, 73). This sets us apart from the majority of previous studies that determined mtDNA copy numbers using only one (32, 38, 54, 59, 72, 74–76) or two mitochondrial genes (13, 49, 77, 78). Additionally, we demonstrated that copy numbers of the ND1 gene exhibit moderate correlation, while the ND4 and CYTB genes show a strong correlation with the composite score, where three genes are moderately correlated each other. Moreover, we confirmed our findings by comparing the composite score among different groups, demonstrating consistently unaltered copy numbers for each individual mitochondrial gene across various study groups. Likewise, we utilized two mitochondrial genes (specifically, ND1 and ND5) for assessing mtDNA oxidative levels, rather than relying on a single mitochondrial gene.

However, the study also had some limitations. First, the sample size was relatively small, which may have hindered the detection of subtle differences and their statistical significance. A larger sample size could potentially reveal significant differences in relative mtDNA-cn and mtDNA-oxi levels at baseline. Second, while PCR is a widely used technique for quantification of these modifications, using more sensitive techniques such as sequencing may lead to more robust findings. Third, our measurements were solely based on blood samples. Employing other specimens, such as brain tissue or saliva, might have yielded different findings. Additionally, making interpretations about brain alterations is limited by the influence of the blood-brain barrier. Fourth, we had data from both the baseline and remitted phases; however, we did not have data from patients who did not achieve remission. Consequently, our study cannot provide information regarding the influence of treatment response. Further studies comparing individuals who respond to antidepressant treatment with those who do not respond will contribute to the knowledge initiated by Fernström et al. (62). Finally, most participants were medicated, even at baseline, so the involvement of medications in the observed changes cannot be entirely ruled out.

In conclusion, the current study revealed significantly increased D-loop methylation levels in individuals with MDD, when compared to the BD group and HCs, suggesting distinct mtDNA modifications in these conditions. However, our findings showed no significant differences in oxidation and copy number of mtDNA. On the other hand, we observed dramatic decreases in relative mtDNA-cn and D-loop methylation during remission, suggesting a potential role of mtDNA variations in the pathophysiology of MDD. To gain a deeper understanding of the different patterns of mtDNA changes between MDD and BD and to assess their significance, it is advisable to conduct future studies with larger sample sizes and consider meta-analyses that pool data from different studies. This approach will provide more robust and comprehensive insights into the involvement of mtDNA in the development and progression of mood disorders.

## Data availability statement

The original contributions presented in the study are included in the article/Supplementary material, further inquiries can be directed to the corresponding authors.

## Ethics statement

The studies involving humans were approved by Koç University Ethics Committee 2022.091.IRB2.016. The studies were conducted in accordance with the local legislation and institutional requirements. The participants provided their written informed consent to participate in this study.

## Author contributions

DC: Conceptualization, Data curation, Formal analysis, Funding acquisition, Investigation, Methodology, Project administration, Resources, Software, Supervision, Validation, Visualization, Writing—original draft, Writing—review & editing. BK: Data curation, Investigation, Methodology, Validation, Writing—review & editing. KT: Data curation, Investigation, Methodology, Validation, Writing—review & editing. IA: Data curation, Investigation, Writing—review & editing. SS: Data curation, Investigation, Writing—review & editing. SG: Conceptualization, Data curation, Formal analysis, Investigation, Methodology, Supervision, Validation, Writing—review & editing.
