# Supplementary material for: Mitochondrial DNA oxidation, methylation, and copy number alterations in major and bipolar depression
Source: Front Psychiatry. 2023 Dec 14;14:1304660. doi: 10.3389/fpsyt.2023.1304660 (PMC10755902; doi:10.3389/fpsyt.2023.1304660)
Supplement: Supplementary file 1 [file Table_1.DOCX]

Supplementary Table 1. Gene sequences of the genes used in the study

| **Gene** | **Gene sequences** | | | **Reference** |
| --- | --- | --- | --- | --- |
|  | **F(5'-3')** | | **R(3'-5')** |  |
| D-loop-methyl | | TAGGAATTAAAGATAGATATTGCGA | ACTCTCCATACATTTAATATTTTCGTC | (Chung et al., 2019a) |
| D-loop-unmethyl | | GGTAGGAATTAAAGATAGATATTGTGA | ACTCTCCATACATTTAATATTTTCATC | (Chung et al., 2019a) |
| PK | | AGCCCAAATGGCCTTGAAG | AGAGACAGAATGCCAGTGAGCT | (Giulivi et al., 2010) |
| CYTB | | CACGATTCTTTACCTTTCACTTCATC | TGATCCCGTTTCGTGCAAG | (Giulivi et al., 2010) |
| ND1 | | CCACCTCTAGCCTAGCCGTTTA | GGGTCATGATGGCAGGAGTAAT | (Giulivi et al., 2010) |
| ND4 | | CCATTCTCCTCCTATCCCTCAAC | CACAATCTGATGTTTTGGTTAAACTATATTT | (Giulivi et al., 2010) |
| ND1 long fragment | | ATGGCCAACCTCCTACTCCT | GATGAGTGTGCCTGCAAAGA | (Czarny et al., 2019) |
| ND1 short fragment | | CCTAAAACCCGCCACATCTA | GCCTAGGTTGAGGTTGACCA | (Czarny et al., 2019) |
| ND5 long fragment | | TCCAACTCATGAGACCCACA | AGGTGATGATGGAGGTGGAG | (Czarny et al., 2019) |
| ND5 short fragment | | AGGCGCTATCACCACTCTGT | TTGGTTGATGCCGATTGTAA | (Czarny et al., 2019) |
